# Routine Orthoptic-led Paediatric Fundus Digital Imaging: Benefits to Patients and Healthcare System

**DOI:** 10.22599/bioj.106

**Published:** 2018-04-24

**Authors:** Laura B. Ramm, Clare Bradbury, Manoj V. Parulekar

**Affiliations:** 1 Eye Department, Birmingham Children’s Hospital NHS Trust, GB

**Keywords:** paediatric, fundus digital imaging, orthoptic-led

## Abstract

**Aims:**

Dilated fundus examinations are a vital, yet time-consuming and sometimes distressing part of paediatric ophthalmology examinations. Limited resources, personnel and time can result in prolonged waiting time and increase risk from delayed diagnosis and treatment. Using a Nikon D80 TopCon TRC-NW6S non-mydriatic fundus camera (TopCon (GB) Ltd, Newbury), we aimed to demonstrate the safety and efficacy of orthoptic-led fundus digital imaging and the potential time and cost benefits to the healthcare system.

**Methods:**

We conducted a retrospective review of all digital fundus images taken over a six month period in 2012 (n = 616, age range 2.1–16.5 years, mean age 8.7 years).

**Results:**

Overall success rate for paediatric fundus digital imaging was 97%. Successful images were achieved in 87% of patients without the need for pupil dilation. Images were taken for a variety of clinical reasons. 45% of patients were discharged immediately, many with copies of photographs to facilitate follow-up with community optometrists.

**Conclusions:**

Orthoptic-led fundus digital imaging is an innovative, speedy, safe and efficient method of documenting fundal appearance, enabling serial documentation of stability/progression of ocular disease. It allows adequate examination of routine patients, freeing up time within busy clinics. Paediatric fundus digital imaging brings a potential positive cost benefit to healthcare systems under pressure, and facilitates skill development for allied health professionals.

## Introduction

Ocular fundus digital imaging is a well-established digital imaging method for documenting the retinal and optic nerves appearance. Clinically, it is widely recognised as standard practice throughout the UK in the retinopathy screening of type II diabetics (NHS 2011; Sharp et al. 2003) and routinely used in conjunction with fluorescein angiographies in the diagnosis and management of adults with diabetic and ischaemic eye disease, choroidal neovascularisation, and papilloedema.

In young babies and infants with retinopathy of prematurity (ROP), retinoblastoma and suspected abusive head trauma (non-accidental injury), fundal appearance is documented with contact retinal imaging (e.g. Retcam, Clarity Medical Systems, CA, USA) and emerging ultra-widefield imaging technologies (e.g. Optos camera, Optos PLC, Dunfermline, UK).

However, the use of such table-mounted digital imaging techniques in paediatric ophthalmology is less common. Literature shows an increased use in paediatric research studies over the past decade (Ribeiro et al. 2007; Jethani et al. 2010; Holl et al. 1998; Andersson and Hellström 2009; Farukhi, Dakkouri and Wang 2006; Salcone, Johnston and VanderVeen 2010; Li, Cheung and Liu 2011); however, clinically, we do not believe this is standard practice in most hospitals.

We aim to present the many advantages of fundus digital imaging becoming a routine part of multi-disciplinary paediatric ophthalmology assessment and the potential positive impact on healthcare systems.

## Methods

We conducted a retrospective clinical notes review of all patients on which digital fundus imaging was attempted in a paediatric out-patient setting over a six-month period in 2012, attending a variety of paediatric eye clinics.

Clinical indication for performing fundus digital imaging was defined as a patient requiring fundus examination (for any reason) who was deemed co-operative enough to sit at table-mounted apparatus. Patients with known ocular pathology were included, and visual acuity was not recorded. Patients on whom digital imaging was attempted but failed were included in the review.

The age of patient, clinical diagnosis, success of photographic imaging, and need for pupil dilation were noted.

The reason for requiring fundus digital imaging was documented, using the following sub-categories:

- Fundus digital imaging used in conjunction with ophthalmologist review, to document ocular pathology/abnormal findings and monitoring of progression of disease (e.g. retinal dystrophies, Coates’ disease, neuro-oncology patients, optic pathway gliomas)
- Fundus photography used in conjunction with ophthalmologist review, to document normal fundus appearance (e.g. screening for papilloedema, or patients with headaches in the absence of suspicious optic discs)
- Fundus photography used to supplement joint orthoptic/optometric examination of newly referred patients, where the input of an ophthalmologist was otherwise not needed (e.g. strabismus or suspected strabismus)
- Fundus photography used to substitute ophthalmologist examination for monitoring of stable ophthalmic conditions (including aphakia, pseudophakia, and glaucoma screening)

Clinical outcomes and subsequent follow-up appointments were documented.

**Figure 1:**
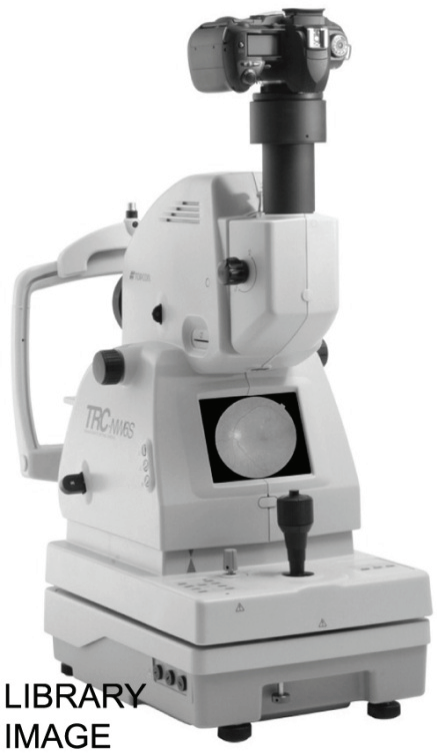
The Nikon D80 TRC-NW6S TopCon (UK) non-mydriatic, table-mounted fundus camera, as used in this study.

All images were 45-degree posterior pole images, centred on the macula, taken by an orthoptist using a Nikon D80 TRC-NW6STopCon (UK) non-mydriatic, table-mounted fundus camera (Figure 1) in dimmed lighting conditions, with parental consent.

Fundus images were printed in colour, and subsequently reviewed ‘virtually’ by a consultant ophthalmologist and documented in patient records.

## Results

Fundus digital imaging was attempted on 616 patients during the study period.

All subjects had complete records and were included in the analysis.

The age of patients ranged from 2.1 to 16.5 years (median 8 years, mean 8.7 years).

**Figure 2:**
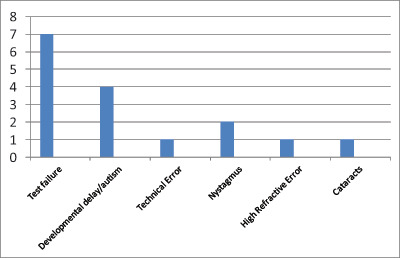
Bar graph showing reasons for unsuccessful imaging in 3% of patients included in this study.

600 (97%) of 616 patients successfully underwent imaging. Sixteen patients (3%) could not be successfully imaged due to test failure, the presence of significant developmental delay or autism, or nystagmus (see Figure 2).

The mean age of patients for whom fundus digital imaging was unsuccessful was 7.8 years.

**Figure 3:**
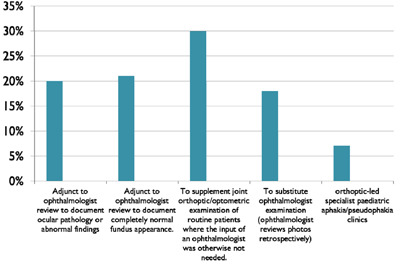
Bar graph showing the clinical indications for paediatric fundus photography in this study.

The clinical indications for fundus digital imaging were varied (see Figure 3).

Of the 600 patients successfully imaged, 522 (87%) did not require pharmaceutical pupil dilation for successful imaging, or for any other part of their ophthalmic work-up.

Of the 600 patients successfully imaged, 270 (45%) were subsequently discharged from the hospital eye service after imaging, with a copy of their fundal images.

## Discussion

Examining the ocular fundus is an important part of a paediatric ophthalmology examination, yet often a dilated eye examination is dreaded by patients, parents and examiners alike.

Fundus examination is vital due to the potentially serious effects of ocular pathology on visual acuity, ocular and general health. Early detection of ocular pathology, and early intervention where possible, maximises the chance of a successful visual outcome and improves quality of life. Confirming the presence of ocular pathology and the subsequent discussion of appropriate aetiologies or interventions is very reassuring for families, whilst confirming the absence of ocular pathology is even more so. Additionally, the medico-legal implications of underlying pathology being undiagnosed are a concern for all clinicians.

It is standard practice that pharmacological preparations are used to dilate pupils, allowing an ophthalmologist or optometrist to visualise the major fundal structures. However, the stinging sensation caused by cycloplegic agents instilled by orthoptic or nursing staff (or immobilisation techniques used) frequently distresses small children, meaning compliance for further medical testing can be limited. Many older patients dislike the blurring of visual acuity caused by cycloplegic drugs, and find this disruptive to schoolwork/after-school activities. The waiting time incurred by using these drops is variable (especially for patients with dark irises) often resulting in appointments which span several hours, and can be perceived as boring for patients and stressful for parents.

A significant majority of patients (87%) in our study did not require dilation for fundus examination. Most patients and families welcome a fundus digital imaging technique, which allows safe, speedy yet accurate examination in a way that is neither distressing nor disruptive for the patient. We believe using a digital imaging method of fundus examination has positive implications on a child’s perception of their visit to the hospital, improves their compliance with further or subsequent testing and aids their relationship with clinicians.

Where visualisation of peripheral retina or cycloplegic refraction was required (10% in this study), an ophthalmologist requested dilating drops. We acknowledge that fundus imaging does not bypass the need for cycloplegic refraction and detailed ophthalmoscopy.

We feel the high success rate of fundus digital imaging (97%) reflects the ease of use of this technique for the paediatric population. For young children, prolonged fundus examination can sometimes be challenging. A major advantage of fundus digital imaging is that images capturing pathology can be assessed in detail by the ophthalmologist, allowing more detailed examination of focal pathology, which can be reviewed by multiple clinicians who can seek further opinions if appropriate.

In a small number of children (3%), fundus digital imaging was attempted but failed. The mean age of patients undergoing unsuccessful photography (7.8 years) was not dissimilar to the mean age of the general study population (8.7 years). This suggests that in cases where imaging was unsuccessful, the patient age was not the reason. The most common reasons for unsuccessful imaging were test failure or the presence of developmental delay/autism. Nystagmus, media opacities, and high refractive errors (e.g. >+/– 15DS) can occasionally be barriers to quality images, but not always.

Although the 45-degree fundus view obtained was narrow in field, in older co-operative patients adequate visualisation with traditional ophthalmoscopy techniques is up to the equator and pre-equatorial retina and is deemed sufficiently safe. Wider view is possible after dilation and with Optos imaging (not included in this study).

There is a theoretical risk of a very peripheral defect being missed using this method where the main purpose is documentation of the posterior pole, and is not advocated as a substitute for a peripheral screening examination.

The clinical scenarios in which we found this imaging technique useful were varied.

Many images were taken at the request of the ophthalmologist, and used in conjunction with a formal ophthalmological assessment. Where ocular pathology was present (20% in this study), common diagnoses included optic nerve disease (papilloedema/atrophy/hypoplasia/anomalous optic discs), retinal lesions (naevus/coloboma), and retinal diseases (dystrophies/macular changes). Serial imaging enables comparison to previous images and assists in documenting progression of disease, which is reassuring to families and can aid education about their child’s condition. Fundus digital imaging is also useful in the absence of clinical pathology (21% in this study), and acts as a pictorial adjunct to medico-legal records.

The most common clinical indication for fundus digital imaging was to supplement the joint orthoptic and/or optometric examination of routine patients with strabismus, amblyopia or refractive error where the input of an ophthalmologist was otherwise not needed (30% in this study). ‘Routine’ was defined as those with no suspected underlying structural abnormality or pathology mentioned in the referral, and were triaged as suitable for that workstream.

Birmingham Children’s Hospital is a large tertiary referral centre with a busy secondary referral orthoptic department, where a significant number of routine cases are managed by orthoptists and optometrists alone. However, it is departmental policy that all patients must have a fundus examination at some point during their care, prior to discharge. Patients with normal monocular visual acuities in the absence of a suspected accommodative esotropia were not routinely refracted by an optometrist (such as patients with pseudosquint, intermittent distance exotropia) and were therefore not dilated. For patients who were not refracted, fundus appearance was still deemed important, and fundus digital imaging allowed adequate fundus visualisation without taking up valuable appointments within the ophthalmology or optometry clinic. This allows the ophthalmologist’s time to be better spent with complex tertiary referrals, which is a more efficient use of their specialist skills.

Patients with reduced vision (monocular or binocular) or suspicion of an accommodative strabismus underwent refraction, and therefore were dilated. Their fundus was visualised traditionally with ophthalmoscopy by an optometrist, and fundus photographs taken upon request only if irregularities found.

For some patients (18% in this study), fundus digital images substituted ophthalmologist examination altogether. This included patients with stable neurological conditions such as hydrocephalus, benign intracranial hypertension, space-occupying lesions, who were not undergoing any active treatment, and those who required initial papilloedema screening. Admittedly, a disadvantage of fundus digital imaging is the inability to assess spontaneous venous pulsation, which can assist in the diagnosis of papilloedema, but is not essential.

Similarly, ophthalmologist review was not required in older patients with port-wine stains who required occasional glaucoma screening, who underwent orthoptic-led intraocular pressure (IOP) monitoring and fundus digital imaging.

Due to our tertiary centre nature, we have significant numbers of patients who are aphakic or pseudophakic following cataract surgery. As part of our service development, over the last five years we have successfully run orthoptic/optometry-led specialist paediatric post-cataract clinics (7% of patients in this study) for children over four years old, who were not undergoing any active treatment (other than amblyopia therapy, contact lens or spectacle use), were considered stable and did not have aphakic glaucoma. We combined standard orthoptic assessment with IOP measurement, fundus digital imaging, and full optometry consultation.

For all of the above scenarios, an ophthalmologist reviewed images taken retrospectively in ‘virtual clinics’, and patients were recalled for ophthalmology review in the event of concerns or if digital imaging was unsuccessful.

Our overall discharge rate for patients included in this study was 45%. We feel fundus digital imaging allows earlier discharge of patients to the care of their community optometrist with copies of their fundus images, allowing their optometrist to monitor for progression of disease and re-referral to hospital eye service should concerns arise.

The entire imaging process used to take a mean time of 12 minutes per patient; however, the image acquisition was just a fraction of this time. Total time included taking parental consent, positioning of patient on equipment, taking the images, printing of documentation and images for clinical notes and manually uploading to safe storage server. However, given recent advancements in our hospital IT systems (with the ability to upload images automatically) this has significantly reduced to a mean time of three minutes. The consultant spends an additional two minutes per patient reviewing the images as a ‘virtual patient’ or administrative exercise. This represents a significant time saving for the multi-disciplinary team, and reduction in financial cost to service commissioners.

Whilst some may consider retinal imaging a technical role, we would argue that successful imaging in a paediatric cohort requires significant paediatric skills (especially for pre-schoolers) and understanding of visual acuity is beneficial. In a department where we are unable to branch out into other well-established adult-based extended role areas (such as glaucoma and retinal disease), orthoptic led fundus digital imaging presents orthoptists with an opportunity for additional skill development and job satisfaction whilst contributing positively to safe and high-quality service delivery.

Since data collection for this study, our reliance on fundus photography has increased and we perform a lot more imaging than in 2012, and despite the initial financial outlay of fundus camera and software, it is considered an essential in our service. In 2016, we performed over 2500 clinical episodes involving fundus photography, representing a twofold increase in activity. With the clinical value of this being recognised, the Trust has invested more in terms of supporting IT and safe storage servers to facilitate this, and we are uploading automatically to the national PACS (Picture Archiving and Communications System) server, which has the advantage of being able to easily transfer images between hospitals. We are currently using a Canon CR-2 Plus AF non-mydriatic camera (Canon UK), with Canon CR-2 Plus retinal imaging software.

Introduction of routine fundus digital imaging improves the efficiency of clinics, aids increased throughput of patients without compromising safety, and assists in the meeting of target times imposed on eye care services. Paediatric fundus digital imaging has a positive impact in terms of patient perception, duration of appointment, and financial cost to the health system.

## Conclusion

Our study supports fundus digital imaging as a useful adjunct to standard assessment in a variety of clinical situations in paediatric ophthalmology. We believe orthoptic-led fundus photography is an innovative, speedy, safe and efficient method of documenting fundal appearance, enabling serial documentation of stability/progression of ocular disease. Success rates for achieving good quality images are high. Fundus digital imaging allows adequate examination of routine patients, freeing up time within busy ophthalmology and optometry clinics.

There is a huge positive cost benefit to healthcare systems under pressure, and development of image aqcuisition skills facilitates development for allied health professionals. In our current practice, we routinely attempt fundus digital imaging from the age of 2–3 years onwards.
